# The impact of relative hypotension on acute kidney injury progression after cardiac surgery: a multicenter retrospective cohort study

**DOI:** 10.1186/s13613-021-00969-4

**Published:** 2021-12-20

**Authors:** Yuki Kotani, Takuo Yoshida, Junji Kumasawa, Jun Kamei, Akihisa Taguchi, Koji Kido, Naoki Yamaguchi, Takayuki Kariya, Masato Nakasone, Noriko Mikami, Takahiro Koga, Izumi Nakayama, Mami Shibata, Tomonao Yoshida, Hiroshi Nashiki, Shinsuke Karatsu, Kazutaka Nogi, Natsuko Tokuhira, Junichi Izawa

**Affiliations:** 1 Department of Intensive Care Medicine, Kameda Medical Center, Kamogawa, Japan; 2 Department of Intensive Care Medicine, Tokyo Women’s Medical University, 8-1 Kawada-cho, Shinjuku-ku, Tokyo, 162-8666 Japan; 3 Department of Critical Care Medicine, Sakai City Medical Center, Sakai, Japan; 4 Emergency and Critical Care Center, Kurashiki Central Hospital, Kurashiki, Japan; 5 Department of Anesthesiology and Critical Care, Kobe City Medical Center General Hospital, Kobe, Japan; 6 Department of Anesthesiology and Intensive Care Medicine, Hiroshima Citizens Hospital, Hiroshima, Japan; 7 Department of Emergency and Critical Care Medicine, Tokyo Bay Urayasu Ichikawa Medical Center, Urayasu, Japan; 8 Intensive Care Department, Yokohama City University Medical Center, Yokohama, Japan; 9 Division of Anesthesiology and Critical Care Medicine, Department of Surgery, Faculty of Medicine, Tottori University, Yonago, Japan; 10 Department of Anesthesiology, Hirosaki University Graduate School of Medicine, Hirosaki, Japan; 11 Intensive Care Unit, Department of Anesthesiology, Jikei University School of Medicine, Tokyo, Japan; 12 Intensive Care Unit, Department of Internal Medicine, Okinawa Chubu Hospital, Okinawa, Japan; 13 Department of Emergency and Critical Care Medicine, Wakayama Medical University, Wakayama, Japan; 14 Department of Emergency Medicine, Hokkaido University Hospital, Sapporo, Japan; 15 Intensive Care Unit, Iwate Prefectural Central Hospital, Morioka, Japan; 16 Department of Intensive Care Medicine, Yokohama City Minato Red Cross Hospital, Yokohama, Japan; 17 Cardiovascular Medicine, Nara Medical University, Kashihara, Japan; 18 Department of Anesthesiology and Intensive Care, Osaka University Graduate School of Medicine, Suita, Japan; 19 Department of Internal Medicine, Okinawa Prefectural Yaeyama Hospital Okinawa, Ishigaki, Japan

**Keywords:** Acute kidney injury, Blood pressure, Cardiac surgery, Cardiogenic shock, Critical care, Hemodynamics

## Abstract

**Background:**

Cardiac surgery is performed worldwide, and acute kidney injury (AKI) following cardiac surgery is a risk factor for mortality. However, the optimal blood pressure target to prevent AKI after cardiac surgery remains unclear. We aimed to investigate whether relative hypotension and other hemodynamic parameters after cardiac surgery are associated with subsequent AKI progression.

**Methods:**

We retrospectively enrolled adult patients admitted to 14 intensive care units after elective cardiac surgery between January and December 2018. We defined mean perfusion pressure (MPP) as the difference between mean arterial pressure (MAP) and central venous pressure (CVP). The main exposure variables were time-weighted-average MPP-deficit (i.e., the percentage difference between preoperative and postoperative MPP) and time spent with MPP-deficit > 20% within the first 24 h. We defined other pressure-related hemodynamic parameters during the initial 24 h as exploratory exposure variables. The primary outcome was AKI progression, defined as one or more AKI stages using Kidney Disease: Improving Global Outcomes’ creatinine and urine output criteria between 24 and 72 h. We used multivariable logistic regression analyses to assess the association between the exposure variables and AKI progression.

**Results:**

Among the 746 patients enrolled, the median time-weighted-average MPP-deficit was 20% [interquartile range (IQR): 10–27%], and the median duration with MPP-deficit > 20% was 12 h (IQR: 3–20 h). One-hundred-and-twenty patients (16.1%) experienced AKI progression. In the multivariable analyses, time-weighted-average MPP-deficit or time spent with MPP-deficit > 20% was not associated with AKI progression [odds ratio (OR): 1.01, 95% confidence interval (95% CI): 0.99–1.03]. Likewise, time spent with MPP-deficit > 20% was not associated with AKI progression (OR: 1.01, 95% CI 0.99–1.04). Among exploratory exposure variables, time-weighted-average CVP, time-weighted-average MPP, and time spent with MPP < 60 mmHg were associated with AKI progression (OR: 1.12, 95% CI 1.05–1.20; OR: 0.97, 95% CI 0.94–0.99; OR: 1.03, 95% CI 1.00–1.06, respectively).

**Conclusions:**

Although higher CVP and lower MPP were associated with AKI progression, relative hypotension was not associated with AKI progression in patients after cardiac surgery. However, these findings were based on exploratory investigation, and further studies for validating them are required.

*Trial Registration* UMIN-CTR, https://www.umin.ac.jp/ctr/index-j.htm, UMIN000037074.

**Supplementary Information:**

The online version contains supplementary material available at 10.1186/s13613-021-00969-4.

## Background

Cardiac surgery is performed worldwide [1–5]. Acute kidney injury (AKI) is a major complication of cardiac surgery. Cardiac surgery-associated AKI (CSA-AKI) occurs in approximately one-third of patients after cardiac surgery and is associated with mortality and morbidity [6–9]. However, there is no effective strategy supported by robust evidence to prevent or treat CSA-AKI [10].

Although blood pressure (BP) is a vital determinant of renal perfusion, the optimal BP target to prevent AKI progression remains unclear for intensive care unit (ICU) patients. Previous randomized controlled trials (RCTs) assessed different absolute mean arterial pressure (MAP) targets [11, 12]. Subgroup analysis of one trial showed that chronic hypertensive patients in the high MAP target group had a lower incidence of AKI [11]. However, these trials showed no difference in mortality between different MAP targets in septic or vasodilatory shock for the overall patient population [11, 12]. Mean perfusion pressure (MPP), which is the difference between MAP and central venous pressure (CVP), can also be used as a surrogate marker for renal perfusion because elevated CVP has been reported as a risk factor for AKI in ICU patients [13, 14]. Although CVP monitoring is readily available in patients after cardiac surgery [15], few studies have assessed the relationship between MPP and AKI in this population [16, 17].

Moreover, relative hypotension, defined as a deficit between the premorbid and achieved BP, has been investigated as a BP target to prevent AKI progression. The Kidney Disease: Improving Global Outcome (KDIGO) guidelines suggest that the optimal BP target may vary according to the premorbid level [18]. Several studies have shown the association of relative hypotension with AKI progression in patients with sepsis or shock [19–21]. Although chronic hypertension is common among cardiac surgery patients [22–24], the only available evidence is a single-center study showing that patients with AKI progression had greater relative hypotension [25].

Accordingly, we conducted a multicenter retrospective cohort study to test whether the relative hypotension using MPP during the first 24 h would be associated with AKI progression between 24 and 72 h among postoperative cardiac surgery patients. We also explored the relationship between other BP-related parameters and subsequent AKI progression.

## Methods

The Blood pressure and Relative Optimal Target after Heart surgery in Epidemiologic Registry (BROTHER) study was a multicenter retrospective cohort study enrolling adult patients after cardiac surgery in 14 ICUs in Japan (UMIN-CTR; trial ID: UMIN000037074). All study patients were followed up until hospital discharge or 30 days after the surgery, whichever came later. The ethics committee of Kameda Medical Center (No. 19-013) and the ethics committees of all other participating hospitals approved this study with an opt-out policy from the patient or proxy. The need for informed consent was waived due to the retrospective nature of this study.

### Participants and data sources/measurement

We consecutively included all adult patients aged 18 years or older who were admitted to one of the ICUs after elective coronary artery bypass grafting (CABG) or valve surgery between January 1st and December 31st in 2018. We excluded (i) patients who were discharged from the ICU within 24 h; (ii) patients who underwent emergency cardiac surgery; (iii) patients on extracorporeal membrane oxygenation, intra-aortic balloon pumping, or ventricular assist device during the first 24 h after ICU admission; (iv) patients with postoperative BP recorded at an interval of longer than 1 h; (v) patients without available BP recordings within 365 days before the surgery; (vi) patients who opted out of this study; and (vii) patients censored for data collection. We defined emergency cardiac surgery as urgent surgery for a life-threatening condition to save life or prevent organ dysfunction. We excluded patients after emergency cardiac surgery because we assumed that detailed information about preoperative blood pressure was often not available, and the calculation of MPP deficiency would be inaccurate.

We collected demographic data, comorbidities, Acute Physiology and Chronic Health Evaluation (APACHE) II score [26], Sequential Organ Failure Assessment score [27], operative characteristics (e.g., type of surgery, surgery time, cardiopulmonary bypass time, transfusion, hemorrhage, and fluid balance), and vasopressor, inotrope, and diuretic use within 24 h after ICU admission. We describe the definitions of variables and details of data collection in Additional file 1: Table S1 and Fig. S1, respectively.

We defined MPP as the difference between MAP and CVP. The preoperative BP was defined as the average of three recent BP readings recorded at least one day apart within 1 year before the surgery. We calculated preoperative MAP as diastolic BP + (systolic BP − diastolic BP)/3. The preoperative CVP was estimated using inferior vena cava data from preoperative echocardiographic findings according to the 2010 American Society of Echocardiography guidelines [28]. When preoperative echocardiography was unavailable, we assumed the mean CVP values stratified for presence or absence of heart disease [29], as previously reported [21, 25]. Additional file 1: Table S2 describes the detailed protocol to determine preoperative CVP.

We collected postoperative MAP and CVP at 1-h intervals during the first 24 h after ICU admission and assumed the CVP value as the average value every 4 h. When there was no available CVP measurement within any 4-h interval, we imputed the CVP value closest to the timepoint.

### Exposure and outcome variables

The primary exposure variable was time-weighted-average MPP-deficit. We defined MPP-deficit as (preoperative MPP − postoperative MPP)/preoperative MPP. We calculated the time-weighted-average MPP-deficit as an aggregate area-under-the-curve divided by 24 h, where the area-under-the-curve was measured as an integrated expression over time using a positive incremental method. When the postoperative MPP was higher than the preoperative MPP, we regarded the MPP-deficit at the timepoint as zero. The secondary exposure variable was time spent with MPP-deficit > 20%, a threshold considered as a significant relative reduction in BP [19, 21]. We also included the following exploratory exposure variables: postoperative time-weighted-average MAP, CVP, MPP, time spent with MAP < 65 mmHg, time spent with MPP < 60 mmHg, time-weighted-average MAP-deficit, and time spent with MAP-deficit > 20%.

The primary outcome variable was AKI progression. We defined AKI progression as an increase of at least one AKI stage between 24 and 72 h compared to the first 24 h. We defined patients with AKI progression as the AKI group and patients without AKI progression as the Non-AKI group. We used both the creatinine and urine output criteria of KDIGO [18]. The intensivist in charge of data collection in each participating hospital clinically determined the preoperative baseline serum creatinine level based on the medical records. The secondary outcome variables included major adverse kidney event (MAKE) within 30 days after the surgery, hospital mortality, renal replacement therapy (RRT) required during ICU stay, fluid balance on the second and third days of ICU admission, new-onset atrial fibrillation during ICU stay, stroke during the hospital stay, and mesenteric ischemia during the hospital stay. We defined MAKE30 as a composite outcome of death, new initiation of RRT, or doubling of serum creatinine from the preoperative level within 30 days after the surgery [30].

### Statistical methods

Among the patients meeting the eligibility criteria, we excluded from our analysis: (i) patients with end-stage kidney disease; (ii) patients with a preoperative serum creatinine > 3.5 mg/dL; (iii) patients who lacked urine output data during the first 24 h after ICU admission; (iv) patients with stage 3 AKI within the first 24 h after ICU admission; (v) patients with < 50% CVP recordings within the first 24 h after ICU admission, and (vi) patients who underwent reoperation during the first 24 h after ICU admission. In all variables except for CVP, we reported the number of missing data if we had missing data and excluded cases with missing data from each analysis. We described how to impute missing postoperative CVP values in “Participants and data source/measurement” section.

We presented categorical variables as numbers with percentages and continuous variables as medians with interquartile range. We compared baseline characteristics and hemodynamic parameters between AKI and Non-AKI groups using the Chi-square test or Fisher’s exact test for categorical variables and the Mann–Whitney *U* test for continuous variables. The impact of all exposure variables on AKI progression was assessed using multivariable logistic regression analyses adjusted for age, APACHE II score, chronic hypertension, preoperative left ventricular ejection fraction, baseline serum creatinine, surgery time, cardiopulmonary bypass (CPB), intraoperative fluid balance, and postoperative red blood cell transfusion within 24 h after ICU admission. We selected these variables based on the clinical relevance and their importance as risk factors for AKI in previous studies [10, 31–35]. The results of multivariable logistic regression models were then plotted using marginal probabilities (average marginal effect) of outcomes across observed ranges of exposure variables to facilitate interpretation.

All analyses were conducted using Stata version 16 (StataCorp, College Station, TX, USA) and EZR (Saitama Medical Center, Jichi Medical University, ver. 1.36), which is a graphical user interface for R (The R Foundation for Statistical Computing, Vienna, Austria). We considered a two-sided *p* value < 0.05 as statistically significant.

We conducted three sensitivity analyses. First, the type of surgery determines the necessity of CPB, which can influence intraoperative management [36] and is associated with postoperative AKI [33]; thus, we performed sensitivity analyses for patients with or without CPB. Second, since less relative hypotension can improve renal perfusion, especially in patients with chronic hypertension, given a potential rightward shift of the curve for organ pressure–flow autoregulation [37], we performed sensitivity analyses for patients with or without chronic hypertension. Third, considering that most previous studies assessing the association of relative hypotension and AKI enrolled only patients with vasopressor support [19, 21, 25], we performed sensitivity analyses for patients with or without vasopressor support. We defined vasopressor support as receiving norepinephrine, epinephrine, dopamine, vasopressin, or phenylephrine during the first 24 h after ICU admission.

## Results

Among 1568 adult patients admitted to the ICUs after CABG or valve surgery between January and December 2018, we registered 870 patients on the BROTHER study. During the data collection period of this study, a pandemic of COVID-19 occurred, which made data collection difficult for some investigators, and we could not enroll 42 patients. After excluding 124 patients, we analyzed 746 patients (Additional file 1: Fig. S2). No patients were lost to follow-up.

Table 1 summarizes the patient characteristics and perioperative management. One-hundred-and-twenty patients (16%) had AKI progression. The median age was 71 years, and most patients (73%) had chronic hypertension. Among the vasopressors and inotropes used in the first 24 h, dobutamine was the most frequently used drug (53%), followed by norepinephrine (40%). The baseline serum creatinine level was higher in the AKI group. Although preoperative MAP was similar between the two groups, preoperative MPP was lower in the AKI group. Additional file 1: Table S3 describes the baseline characteristics and perioperative management in detail.

**Table 1:** Patient characteristics and perioperative management

	All *N* = 746	AKI group *N* = 120	Non-AKI group *N* = 626	*p* value
Age, years	71 [63–77]	72 [64–77]	70 [63–77]	0.58
Males, *n* (%)	464 (62)	73 (61)	391 (63)	0.76
Chronic hypertension, *n* (%)	546 (73)	88 (73)	458 (73)	1.0
Previous cardiac surgery, *n* (%)	74 (9.9)	18 (15)	56 (8.9)	0.047
LVEF, *n* (%)				0.72
> 50%	596 (80)	95 (79)	50.1 (80)	
35–50%	120 (16)	22 (18)	98 (16)	
20–34%	28 (3.8)	3 (2.5)	25 (4.0)	
< 20%	2 (0.3)	0 (0)	2 (0.3)	
Baseline serum creatinine, mg/dL	0.85 [0.70–1.03]	0.90 [0.77–1.08]	0.83 [0.70–1.01]	0.0051
Baseline MPP, mmHg	80 [74–88]	78 [71–85]	81 [74–88]	0.020
Baseline MAP, mmHg	85 [78–92]	83 [77–89]	85 [78–92]	0.089
Baseline CVP, mmHg	3 [3–6]	3 [3–6]	3 [3–6]	0.0013
Type of surgery, *n* (%)				
CABG	257 (35)	37 (30.8)	220 (35.1)	0.40
Mitral valve	245 (33)	51 (42.5)	194 (31.0)	0.019
Aortic valve	358 (48)	60 (50.0)	298 (47.6)	0.69
Tricuspid valve	138 (19)	31 (25.8)	107 (17.1)	0.029
Pulmonary valve	9 (1.2)	0	9 (1.4)	0.37
Surgery time, minutes	342 [277–428]	351 [300–443]	340 [273–426]	0.25
Cardiopulmonary bypass	650 (87)	106 (88)	544 (87)	0.77
Cardiopulmonary bypass time, minutes^a^	178 [135–231]	199 [147–238]	174 [134–229]	0.040
Intraoperative fluid balance, mL	2297 [1212–3335]	2351 [1317–3208]	2259 [1206–3347]	0.83
APACHE II score	13 [11–15]	13 [11–15]	13 [11–15]	0.098
Vasopressors and inotropes within the first 24 h after ICU admission				
Norepinephrine, *n* (%)	295 (40)	45 (38)	250 (40)	0.68
Maximal dose^b^, µg/kg/min	0.08 [0.05–0.14]	0.06 [0.04–0.12]	0.08 [0.05–0.14]	0.51
Epinephrine, *n* (%)	6 (0.8)	0 (0)	6 (1.0)	0.60
Maximal dose^b^, µg/kg/min	0.05 [0.03–0.07]	0	0.05 [0.03–0.07]	
Dopamine, *n* (%)	128 (17)	16 (13)	112 (18)	0.29
Maximal dose^b^, µg/kg/min	3.1 [1.9–4.1]	3.6 [1.9–4.8]	3.0 [1.9–4.1]	0.52
Vasopressin, *n* (%)	31 (4.2)	2 (1.7)	29 (4.6)	0.21
Phenylephrine, *n* (%)	10 (1.3)	0	10 (1.6)	0.38
Dobutamine, *n* (%)	406 (53)	74 (62)	332 (53)	0.089
Maximal dose^b^, µg/kg/min	2.6 [1.7–3.5]	3.0 [1.6–3.7]	2.6 [1.7–3.5]	0.31
PDE inhibitors, *n* (%)	135 (18)	31 (26)	104 (17)	0.020
Blood products within the first 24 h after ICU admission, *n* (%)				
Red blood cells, units	173 (23)	23 (19)	150 (24)	0.29

*IQR* interquartile range, *APACHE* Acute Physiology and Chronic Health Evaluation, *SOFA* Sequential Organ Failure Assessment, *AIDS* acquired immunodeficiency syndrome, *LVEF* left ventricular ejection fraction, *ICU* intensive care unit, CABG coronary artery bypass grafting, *PDE* phosphodiesterase

^a^The denominator was patients in whom cardiopulmonary bypass surgery was conducted

^b^The denominator was patients in whom each vasoactive agent was given during the first 24 h after ICU admission

**Fig. 1:**
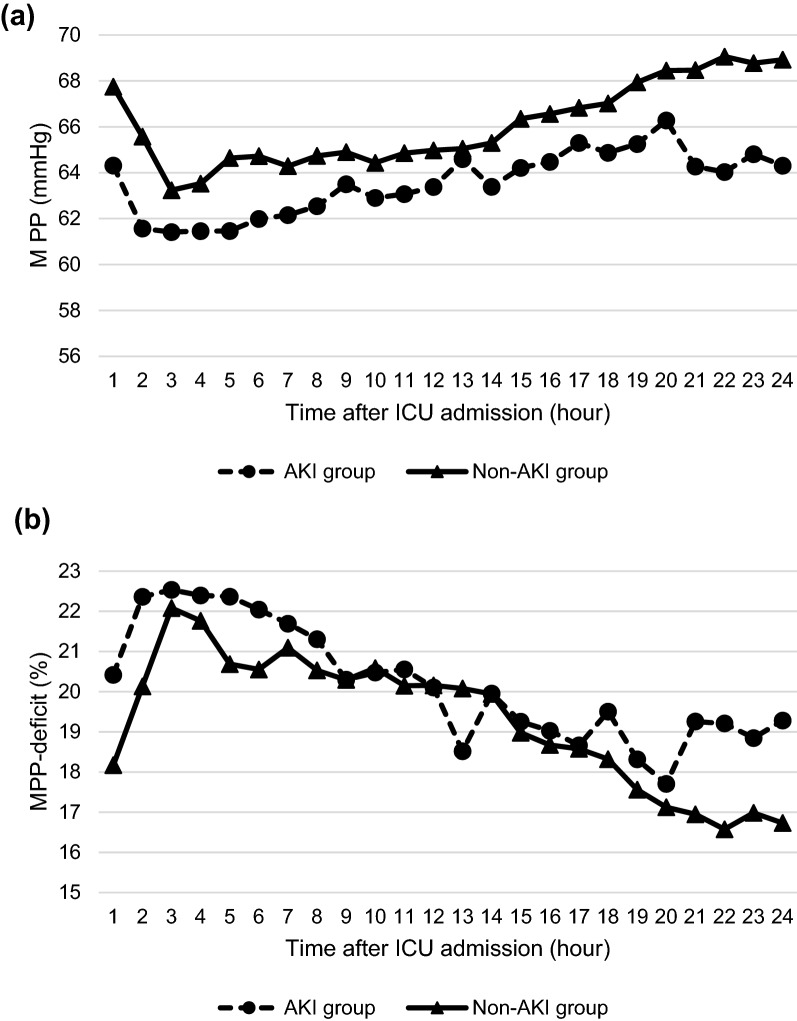
Achieved mean perfusion pressure (**a**) and mean perfusion pressure-deficit (**b**) during the first 24 h after intensive care unit admission. *AKI* acute kidney injury, *ICU* intensive care unit, *MPP* mean perfusion pressure

Table 2 compares the postoperative hemodynamic parameters and exposure variables between the AKI and Non-AKI groups. CVP was missing for 198 (4.4%) out of 4,476 timepoints. While both groups achieved similar time-weighted-average MAP, the AKI group achieved a higher CVP, which resulted in a lower achieved MPP and more prolonged duration with MPP < 60 mmHg in the AKI group. Time-weighted-average MPP-deficit and time spent with MPP-deficit > 20% were comparable between the two groups (Fig. 1). Although time spent with MAP < 65 mmHg was statistically significantly longer in the AKI group, the numerical difference was slight. Time-weighted-average MAP-deficit and time spent with MAP-deficit > 20% were similar between the two groups (Additional file 1: Fig. S3). Table 3 describes the clinical outcomes. Seven patients (0.9%) experienced MAKE30, and hospital mortality occurred in four patients (0.5%).

**Table 2:** Hemodynamic parameters and exposure variables

	All *N* = 746	AKI group *N* = 120	Non-AKI group *N* = 626	*p* value
Achieved MPP (time-weighted average), mmHg	65 [60–71]	63 [59–69]	66 [60–71]	0.0047
Achieved MAP (time-weighted average), mmHg	74 [69–79]	72 [68–78]	74 [69–79]	0.051
Achieved CVP (time-weighted average), mmHg	8.4 [6.7–11]	9.4 [7.2–11]	8.3 [6.6–10]	0.0048
Time spent with MPP < 60 mmHg, h	6 [2–13]	7 [4–13]	5 [2–12]	0.0016
MPP-deficit (time-weighted average), %	20 [10–27]	22 [11–28]	19 [10–27]	0.41
Time spent with MPP-deficit > 20%, h	12 [3–20]	13 [4–20]	11 [3–20]	0.38
Time spent with MAP < 65 mmHg, h	2 [0–6]	3 [1–7]	2 [0–6]	0.0090
MAP-deficit (time-weighted average), %	14 [6.2–21]	14 [7.1–21]	14 [6.0–21]	0.54
Time spent with MAP-deficit > 20%, h	5 [1–14]	5 [1–15]	5 [1–14]	0.52

*MAP* mean arterial pressure, *CVP* central venous pressure, *MPP* mean perfusion pressure

**Table 3:** Clinical outcomes

	All *N* = 746
AKI progression between 24 and 72 h after ICU admission, *n* (%)	120 (16.1)
MAKE30, *n* (%)	7 (0.9)
Hospital mortality, *n* (%)	4 (0.5)
Need for renal replacement therapy during ICU stay, *n* (%)	5 (0.7)
Fluid balance on the second day of ICU stay, mL	530.5 [− 86.3–1271.8]
Fluid balance on the third day of ICU stay^a^, mL	− 429.0 [− 1240.0–100.0]
New-onset atrial fibrillation during ICU stay^b^, *n* (%)	86 (14.9)
Stroke during hospital stay, *n* (%)	12 (1.6)
Mesenteric ischemia during hospital stay, *n* (%)	0

*AKI* acute kidney injury, *MAKE* major adverse kidney event, *ICU* intensive care unit, *IQR* interquartile range

^a^The denominator was patients who stayed in the ICU over 3 calendar days or more (*N* = 734)

^b^The denominator was patients without a history of atrial fibrillation before ICU admission (*N* = 577)

**Fig. 2:**
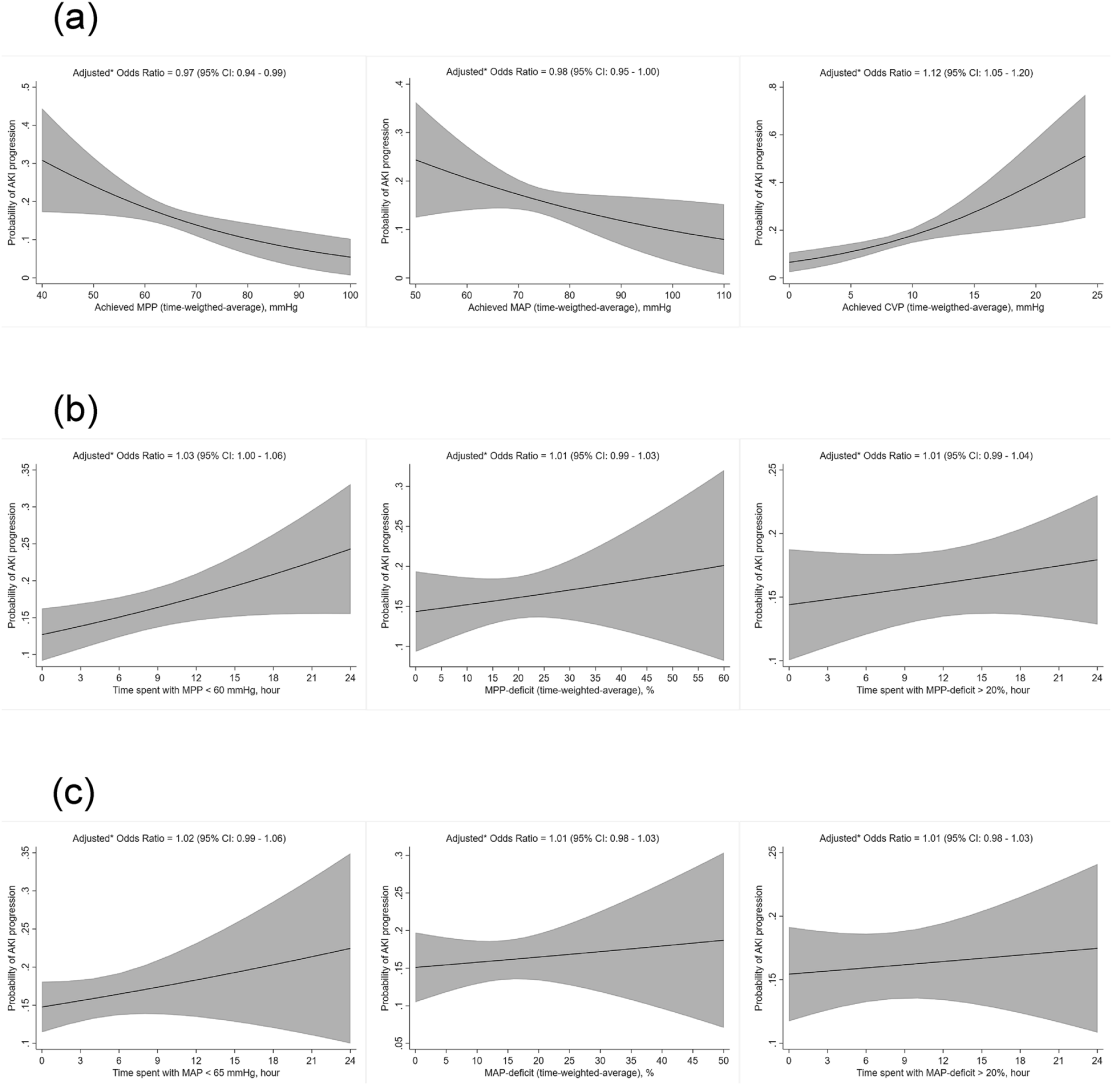
Predicted marginal probabilities with 95% confidence intervals for acute kidney injury progression versus exposure variables. **a** Achieved blood pressure. **b** Mean perfusion pressure. **c** Mean arterial pressure. *AKI* acute kidney injury, *MAP* mean arterial pressure, *CVP* central venous pressure, *MPP* mean perfusion pressure, *CI* confidence interval

Multivariable logistic regression analyses (Additional file 1: Table S4 and Fig. 2) showed that, for every percent increase in time-weighted-average MPP-deficit, the adjusted odds ratio (OR) for AKI progression was 1.01 [95% confidence interval (CI), 0.99–1.03; *p* = 0.46]. Similarly, for every hour increase in time spent with MPP-deficit > 20%, the adjusted OR for AKI progression was 1.01 (95% CI 0.99–1.04; *p* = 0.38). Among the exploratory exposure variables, time-weighted-average CVP (adjusted OR: 1.12, 95% CI 1.05–1.20; *p* = 0.0013), time-weighted-average MPP (adjusted OR: 0.97, 95% CI 0.94–0.99; *p* = 0.0078), and time spent with MPP < 60 mmHg (adjusted OR: 1.03, 95% CI 1.00–1.06; *p* = 0.024) were statistically significantly associated with AKI progression.

The results of the sensitivity analyses are presented in Additional file 1: Table S5. Again, we found results similar to those of the main analyses.

## Discussion

### Key findings

This multicenter retrospective cohort study investigated whether relative hypotension and other BP-related hemodynamic parameters were associated with subsequent AKI progression in adult patients after elective cardiac surgery. Among 746 patients eligible for this analysis, the occurrence of AKI progression was 16%. We observed that relative hypotension was not associated with AKI progression, while higher CVP, lower MPP, and time spent with MPP < 60 mmHg were associated with AKI progression among exploratory exposure variables.

### Relationship with previous studies

Recent studies have assessed the relationship between relative hypotension and AKI progression in critically ill patients [19–21, 25]. Among those, the only multicenter study, which enrolled 302 vasopressor-dependent patients with vasodilatory shock, showed that relative hypotension using MPP was an independent risk factor for AKI progression after adjusting for relevant confounders [21]. The only evidence for cardiac surgery patients showed that those who developed AKI progression had greater relative hypotension than those who did not [25]. However, this was a small single-center study that included only vasopressor-dependent patients and lacked multivariable analyses.

Our results were inconsistent with previous studies [21, 25]. The multivariable analyses found that relative hypotension was not associated with AKI progression, although lower postoperative MPP was significantly associated with AKI progression. The sensitivity analyses, including patients with or without vasopressor support, also showed similar results. Greater relative hypotension was caused by higher preoperative or lower postoperative MPP, while smaller relative hypotension was caused by lower preoperative or higher postoperative MPP. Since previous studies [16, 17, 38] and our study have consistently shown that lower postoperative MPP is a risk factor of AKI, the non-linear relationship between preoperative MPP and AKI progression could weaken the association between relative hypotension and AKI progression. Higher preoperative MPP could increase the risk of AKI due to the right shift in the lower pressure limit of renal autoregulation in the presence of chronic hypertension [14]. On the contrary, lower preoperative MPP may also be a risk factor for postoperative AKI, especially in cardiac surgery patients. For example, chronic heart failure with reduced ejection fraction could result in low baseline MAP and is a risk factor for CSA-AKI [32, 39]. Patients with pulmonary hypertension and right heart failure could have high preoperative CVP and are at an increased risk of developing AKI [40]. Therefore, the effect of lower preoperative MPP on AKI among cardiac surgery patients may contribute to the different relationships of relative hypotension with AKI progression between cardiac surgery patients and other patient groups [21]. We report a lower preoperative MPP in our cohort (80 mmHg) than the previous study enrolling cardiac surgery patients (89 mmHg) [25], which may imply that we enrolled more patients with low preoperative MPP, resulting in a weaker correlation between relative hypotension and AKI progression. Moreover, we conducted multivariable logistic regression analyses, ensuring the robustness of our findings.

During the acute phase after cardiac surgery, routine CVP monitoring has helped guide hemodynamic management, such as detecting acute pericardial tamponade or right heart failure [41]. In addition to the utility in clinical practice, recent studies reported that elevated CVP is a prognostic indicator of poor outcomes in ICU patients. A meta-analysis reported the association of higher CVP with mortality and AKI among critically ill patients [13]. Observational studies showed that elevated CVP was a risk factor for mortality or AKI in cardiac surgery patients [42–44]. In line with these reports, we showed that increased CVP was associated with AKI progression. We also found that lower MPP was associated with AKI progression. Thus, out of the two components of MPP, i.e., MAP and CVP, CVP would substantially influence AKI progression in patients after cardiac surgery. As for the septic patients, CVP monitoring is not routine because CVP has been suggested as a poor indicator of fluid status. The previous large RCT for septic patients could not evaluate MPP [11]. However, a small observational study suggested that MPP might be associated with AKI [20]. Thus, in future studies for septic patients, reevaluation of CVP monitoring might be of value for preventing AKI progression.

### Significance and implications

We report that although relative hypotension was not associated with AKI progression among patients after elective cardiac surgery, higher CVP and lower MPP were associated. Based on these results, we do not support adjusting MPP targets to the premorbid BP to prevent AKI progression after cardiac surgery. Since sequential CVP measurement is generally available using pulmonary artery catheters or central venous catheters after cardiac surgery, clinicians should pay more attention to high CVP values to identify patients at risk of AKI progression. In patients with elevated CVP, clinicians may consider avoiding interventions that can further increase the CVP value, such as excessive fluid resuscitation. Future studies are necessary to investigate whether lowering CVP values, such as with early diuretics administration, would decrease AKI progression among patients after cardiac surgery.

### Strengths and limitations

This study is the first multicenter study assessing the relationship between relative hypotension and AKI progression in cardiac surgery patients using multivariable analyses adjusted for clinically relevant confounders. We had the largest sample size among the studies assessing relative hypotension and AKI in critically ill patients. In addition, there were no missing data in postoperative MAP at 1-h intervals, ensuring detailed and accurate BP data collection.

Our study has several limitations. First, time-weighted-average BP may underestimate profound hypotension within a short time. However, we used the time spent with BP under a certain threshold as exposure variables to overcome the shortcoming of time-weighted-average BP. In addition, the associations of the two measures of exposure variable with AKI progression were consistent in all exposure variables, showing the robustness of the results. Second, we included some intraoperative confounders into the multivariable analyses on postoperative AKI progression. However, we did not collect BP data during the cardiac surgery. In addition, patients with AKI progression had longer cardiopulmonary bypass time. We may be missing some of the effects of intraoperative management. Future studies should deal with intraoperative variables in more detail. Third, as an observational cohort study, we could not control unmeasured or unknown confounders that may have influenced the results.

## Conclusions

We found that relative hypotension is not associated with AKI progression, while higher CVP and lower MPP are associated with AKI progression in patients after cardiac surgery. However, these findings were based on exploratory investigation, and further studies for validating them are required.

## Supplementary Information

**Additional file 1: Table S1.** Data collection and definitions of variables and outcomes. **Table S2.** The protocol to estimate preoperative central venous pressure. **Table S3.** Additional information on baseline characteristics and perioperative management. **Table S4.** Multivariable logistic regression analyses for acute kidney injury progression between 24 and 72 h after intensive care unit admission. **Table S5.** Sensitivity analyses for acute kidney injury progression between 24 and 72 h after intensive care unit admission. **Figure S1.** Details of data collection. **Figure S2.** Patient flow diagram. **Figure S3.** Achieved mean arterial pressure (a) and mean arterial pressure-deficit (b) during the first 24 h after intensive care unit admission.

## Data Availability

The data that support the findings of this study are available from the corresponding author, TaY, upon reasonable request.

## Abbreviations

AKI: Acute kidney injury
APACHE: Acute Physiology and Chronic Health Evaluation
BROTHER: Blood pressure and Relative Optimal Target after Heart surgery in Epidemiologic Registry
CABG: Coronary artery bypass grafting
CI: Confidence interval
CPB: Cardiopulmonary bypass
CVP: Central venous pressure
ECMO: Extracorporeal membrane oxygenation
ICU: Intensive care unit
KDIGO: Kidney Disease: Improving Global Outcomes
MAP: Mean arterial pressure
MAKE: Major adverse kidney event
MPP: Mean perfusion pressure
OR: Odds ratio
RRT: Renal replacement therapy
