# Supplementary material for: The impact of relative hypotension on acute kidney injury progression after cardiac surgery: a multicenter retrospective cohort study
Source: Ann Intensive Care. 2021 Dec 20;11:178. doi: 10.1186/s13613-021-00969-4 (PMC8686086; doi:10.1186/s13613-021-00969-4)
Supplement: Supplementary file 1 — Additional file 1: Table S1. Data collection and definitions of variables and outcomes. Table S2. The protocol to estimate preoperative central venous pressure. Table S3. Additional information on baseline characteristics and perioperative management. Table S4. Multivariable logistic regression analyses for acute kidney injury progression between 24 and 72 h after intensive care unit admission. Table S5. Sensitivity analyses for acute kidney injury progression between 24 and 72 h after intensive care unit admission. Figure S1. Details of data collection. Figure S2. Patient flow diagram. Figure S3. Achieved mean arterial pressure (a) and mean arterial pressure-deficit (b) during the first 24 h after intensive care unit admission. [file 13613_2021_969_MOESM1_ESM.docx]

**The impact of relative hypotension on acute kidney injury progression**

**after cardiac surgery: a multicenter retrospective cohort study**

Additional file 1

Table of contents

[Table S1. Data collection and definitions of variables and outcomes. 2](#_Toc77863011)

[Table S2: The protocol to estimate preoperative CVP 5](#_Toc77863012)

[Table S3: Additional information on baseline characteristics and perioperative management 6](#_Toc77863013)

[Table S4: Multivariable logistic regression analyses for AKI progression between 24 and 72 hours after ICU admission 7](#_Toc77863014)

[Table S5: Sensitivity analyses for AKI progression between 24 and 72 hours after ICU admission 8](#_Toc77863015)

[Figure S1: Details of data collection 11](#_Toc77863016)

[Figure S2: Patient flow diagram 12](#_Toc77863017)

[Figure S3: Achieved MAP (a) and MAP-deficit (b) during the first 24 hours after ICU admission 13](#_Toc77863018)

# Table S1. Data collection and definitions of variables and outcomes.

| Demographics | Age, sex, height, body weight, and date of ICU and hospital admission. |
| --- | --- |
| APACHE II score (1) | Calculated from the data within the first 24 hours after ICU admission. |
| SOFA score (2) | Calculated from the data within the first 24 hours after ICU admission. |
| Past medical histories | Chronic hypertension, diabetes mellitus, dyslipidemia, ischemic heart disease, atrial fibrillation, chronic heart failure, chronic lung disease, liver cirrhosis, liver failure, leukemia/myeloma, lymphoma, immunosuppression, chronic kidney disease, and previous cardiac surgery. |
| Chronic hypertension | Based on previous medications. |
| Diabetes mellitus | Based on previous medications. |
| Dyslipidemia | Based on previous statin therapy. |
| Ischemic heart disease | Based on the history of angina pectoralis or myocardial infarction. |
| Atrial fibrillation | Based on medical record, preoperative electrocardiogram, or echocardiography. |
| Chronic heart failure | Having New York Heart Association Class IV (3). |
| Chronic lung disease | Documented chronic hypoxia, hypercapnia, secondary polycythemia, severe pulmonary hypertension (>40 mmHg), or respirator dependency. |
| Liver cirrhosis | Biopsy proven cirrhosis and documented portal hypertension. |
| Liver failure | Having episodes of jaundice, ascites, upper gastrointestinal bleeding, or hepatic encephalopathy in addition to liver cirrhosis defined above. |
| Leukemia/myeloma | Having the history of leukemia (including both acute/chronic and myeloblastic/lymphoblastic) or myeloma within five years before the surgery. |
| Lymphoma | Having the history of lymphoma within five years before the surgery. |
| Immunosuppression | The patient has received immunosuppressants, chemotherapy, radiation, corticosteroids (equivalent of prednisolone 0.375 mg/kg/day or more) within the six months before the surgery. |
| Chronic kidney disease | Determined based on baseline serum creatinine. |
| Previous cardiac surgery | History of coronary artery bypass grafting or valve surgery. |
| Left ventricular ejection fraction | Based on the preoperative echocardiographic findings. |
| Diameter and respiratory change of inferior vena cava | Based on the preoperative echocardiographic findings. The respiratory change in inferior vena cava is defined as >50% collapsibility. |
| Baseline serum creatinine | The intensivist in charge of data collection in each participating hospital clinically determined preoperative baseline serum creatinine level from the patients’ medical records. |
| Preoperative blood pressure | The average of three recent blood pressure readings recorded at least one day apart within one year before the surgery. Mean arterial pressure was calculated by diastolic blood pressure + (systolic blood pressure – diastolic blood pressure)/3. |
| Intraoperative information | Type of surgery, surgery time, surgery time, cardiopulmonary bypass time, intraoperative intravenous fluid, transfusion during the surgery, intraoperative hemorrhage, and intraoperative fluid balance. |
| Type of surgery | Coronary artery bypass grafting and heart valve surgery. Any transcatheter valve surgery was excluded. |
| Clinical outcomes | Fluid balance on the second and third day of ICU stay, hospital mortality, RRT required during ICU stay, serum creatinine and RRT dependence on the 30^th^ day after ICU admission, new-onset atrial fibrillation during ICU stay, mesenteric ischemia during hospital stay, and stroke during hospital stay. |
| Postoperative blood pressure and central venous pressure | Systolic, mean, and diastolic blood pressure and central venous pressure at one-hour interval. |
| Vasopressors and inotropes | Norepinephrine, dobutamine, epinephrine, dopamine, PDE inhibitors, vasopressin, and phenylephrine. |
| Diuretics | Loop, potassium-sparing, acetazolamide, tolvaptan, and carperitide. |
| Transfusion | Red blood cells, fresh frozen plasma, and platelets |
| Reoperation | Reoperation due to massive hemorrhage |
| KDIGO staging | serum creatinine and urine output based on the KDIGO classification (4) |

ICU: intensive care unit; APACHE: acute physiology and chronic health evaluation; SOFA: sequential organ failure assessment; RRT: renal replacement therapy; PDE:　phosphodiesterase; KDIGO: Kidney Disease: Improving Global Outcomes

**References**

1. Knaus WA, Draper EA, Wagner DP, Zimmerman JE. APACHE II: a severity of disease classification system. Critical care medicine. 1985;13(10):818-29.

2. Vincent JL, Moreno R, Takala J, Willatts S, De Mendonça A, Bruining H, et al. The SOFA (Sepsis-related Organ Failure Assessment) score to describe organ dysfunction/failure. On behalf of the Working Group on Sepsis-Related Problems of the European Society of Intensive Care Medicine. Intensive care medicine. 1996;22(7):707-10.

3. THE CLASSIFICATION OF CARDIAC DIAGNOSIS. Journal of the American Medical Association. 1921;77(18):1414-5.

4. KDIGO. KDIGO clinical practice guideline for acute kidney injury. Kidney Int Suppl. 2012;2:1-138.

# Table S2: The protocol to estimate preoperative CVP

| 1. We estimated preoperative CVP based on inferior vena cava (IVC) diameter and collapsibility at the preoperative echocardiography.  - Preoperative CVP = 3 mmHg if IVC diameter $\leq$21 mm with >50% collapsibility. - Preoperative CVP = 8 mmHg if IVC diameter >21 mm with >50% collapsibility, or if IVC diameter $\leq$21 mm with $\leq$50% collapsibility. - Preoperative CVP = 15 mmHg if IVC diameter >21 mm with $\leq$50% collapsibility. |
| --- |
| 1. If preoperative echocardiography is not available, we estimated preoperative CVP as 8 mmHg in patients undergoing valve surgery considering that there is at least moderate valvular dysfunction. |
| 1. If there was no preoperative echocardiography and the patient received coronary artery bypass grafting without valve surgery, we estimated the preoperative CVP of 6 mmHg. |

CVP: central venous pressure

# Table S3: Additional information on baseline characteristics and perioperative management

|  | All  N = 746 | AKI group  N = 120 | Non-AKI group  N = 626 | p value |
| --- | --- | --- | --- | --- |
| Body mass index, kg/m2 | 23 [21-26] | 24 [22-26] | 23 [21-26] | 0.56 |
| Diabetes mellitus, n (%) | 179 (24) | 29 (24) | 150 (24) | 1.0 |
| Dyslipidemia | 330 (44) | 47 (39) | 283 (45) | 0.23 |
| Ischemic heart disease, n (%) | 275 (37) | 46 (38) | 229 (37) | 0.76 |
| Chronic heart failure, n (%) | 20 (2.7) | 3 (2.5) | 17 (2.7) | 1.0 |
| Chronic kidney disease, n (%) | 315 (42) | 61 (51) | 254 (41) | 0.043 |
| Liver cirrhosis | 2 (0.3) | 1 (0.8) | 1 (0.2) | 0.30 |
| Liver failure | 1 (0.1) | 0 (0) | 1 (0.1) | 1.0 |
| Leukemia/myeloma | 3 (0.4) | 0 (0) | 3 (0.5) | 1.0 |
| Lymphoma | 1 (0.1) | 0 (0) | 1 (0.2) | 1.0 |
| Immunosuppression | 3 (0.4) | 0 (0) | 3 (0.5) | 1.0 |
| Intraoperative RBC transfusion, units | 4 [0-6] | 4 [0-8] | 4 [0-6] | 0.018 |
| Intraoperative FFP transfusion, units | 4 [0-8] | 4 [0-8] | 4 [0-8] | 0.16 |
| Intraoperative platelet transfusion, units | 0 [0-10] | 0 [0-20] | 0 [0-10] | 0.17 |
| Intraoperative hemorrhage, mL | 300 [150-640] | 330 [160-583] | 300 [150-657] | 0.76 |
| SOFA score | 7 [6-8] | 7 [6-8] | 7 [6-8] | 0.23 |
| Diuretics within the first 24 hours after ICU admission, n (%) | | | | |
| Loop | 236 (32) | 28 (23) | 208 (33) | 0.033 |
| Potassium-sparing | 42 (5.6) | 7 (5.8) | 35 (5.6) | 0.83 |
| Tolvaptan | 25 (3.4) | 3 (2.5) | 22 (3.5) | 0.78 |
| Carperitide | 164 (22) | 40 (33) | 124 (20) | 0.0017 |
| Blood products within the first 24 hours after ICU admission, n (%) | | | | |
| Fresh frozen plasma, units | 236 (32) | 36 (30) | 200 (32) | 0.75 |
| Platelets, units | 100 (13) | 17 (14) | 83 (13) | 0.77 |

AKI: acute kidney injury; RBC: red blood cell; FFP: fresh frozen plasma; ICU: intensive care unit

# Table S4: Multivariable logistic regression analyses for AKI progression between 24 and 72 hours after ICU admission

|  | Adjusted OR  (95% CI) | p value |
| --- | --- | --- |
| Achieved MPP (time-weighted-average), mmHg | 0.97 (0.94-0.99) | 0.0078 |
| Achieved MAP (time-weighted-average), mmHg | 0.98 (0.95-1.00) | 0.10 |
| Achieved CVP (time-weighted-average), mmHg | 1.12 (1.05-1.20) | 0.0013 |
|  |  |  |
| Time spent with MPP <60 mmHg, hour | 1.03 (1.00-1.06) | 0.024 |
| MPP-deficit (time-weighted average), % | 1.01 (0.99-1.03) | 0.46 |
| Time spent with MPP-deficit >20%, hour | 1.01 (0.99-1.04) | 0.38 |
|  |  |  |
| Time spent with MAP <65 mmHg, hour | 1.02 (0.99-1.06) | 0.24 |
| MAP-deficit (time-weighted-average), % | 1.01 (0.98-1.03) | 0.63 |
| Time spent with MAP-deficit >20%, hour | 1.01 (0.98-1.03) | 0.64 |

Adjusted on age, APACHE II score, chronic hypertension, preoperative left ventricular ejection fraction, baseline serum creatinine, surgery time, cardiopulmonary bypass, intraoperative fluid balance, and postoperative red blood cell transfusion within 24 hours after ICU admission.

OR: odds ratio; CI: confidence interval; AKI: acute kidney injury; ICU: intensive care unit; MPP: mean perfusion pressure; MAP: mean arterial pressure

# Table S5: Sensitivity analyses for AKI progression between 24 and 72 hours after ICU admission

|  | Adjusted OR  (95% CI) | p for interaction |
| --- | --- | --- |
| Achieved MPP (time-weighted-average), mmHg | | |
| Overall | 0.97 (0.94-0.99) |  |
| CPB + | 0.96 (0.94-0.99) | 0.084 |
| CPB - | 1.01 (0.91-1.13) |  |
| Chronic hypertension + | 0.97 (0.94-0.997) | 0.82 |
| Chronic hypertension - | 0.96 (0.91-1.01) |  |
| Vasopressor + | 0.95 (0.91-0.99) | 0.41 |
| Vasopressor - | 0.99 (0.95-1.02) |  |
| Achieved MAP (time-weighted-average), mmHg | | |
| Overall | 0.98 (0.95-1.00) |  |
| CPB + | 0.97 (0.95-1.00) | 0.15 |
| CPB - | 1.01 (0.91-1.12) |  |
| Chronic hypertension + | 0.98 (0.95-1.01) | 0.81 |
| Chronic hypertension - | 0.97 (0.921.03) |  |
| Vasopressor + | 0.97 (0.93-1.01) | 0.79 |
| Vasopressor - | 0.99 (0.95-1.03) |  |
| Achieved CVP (time-weighted-average), mmHg | | |
| Overall | 1.12 (1.05-1.20) |  |
| CPB + | 1.14 (1.05-1.23) | 0.21 |
| CPB - | 0.98 (0.75-1.29) |  |
| Chronic hypertension + | 1.13 (1.04-1.23) | 0.99 |
| Chronic hypertension - | 1.13 (0.98-1.29) |  |
| Vasopressor + | 1.22 (1.09-1.36) | 0.16 |
| Vasopressor - | 1.04 (0.95-1.15) |  |
| Time spent with MPP <60 mmHg, hour | | |
| Overall | 1.03 (1.00-1.06) |  |
| CPB + | 1.04 (1.01-1.07) | 0.14 |
| CPB - | 0.97 (0.84-1.13) |  |
| Chronic hypertension + | 1.03 (0.998-1.07) | 0.82 |
| Chronic hypertension - | 1.04 (0.98-1.11) |  |
| Vasopressor + | 1.05 (1.00-1.10) | 0.68 |
| Vasopressor - | 1.02 (0.98-1.06) |  |
| MAP-deficit (time-weighted-average), % |  |  |
| Overall | 1.01 (0.98-1.03) |  |
| CPB + | 1.01 (0.99-1.04) | 0.11 |
| CPB - | 0.97 (0.89-1.06) |  |
| Chronic hypertension + | 1.01 (0.98-1.03) | 0.68 |
| Chronic hypertension - | 1.00 (0.95-1.04) |  |
| Vasopressor + | 1.01 (0.97-1.04) | 0.90 |
| Vasopressor - | 1.00 (0.98-1.04) |  |
| MPP-deficit (time-weighted-average), % |  |  |
| Overall | 1.01 (0.99-1.03) |  |
| CPB + | 1.01 (0.99-1.03) | 0.055 |
| CPB - | 0.97 (0.89-1.04) |  |
| Chronic hypertension + | 1.01 (0.99-1.03) | 0.90 |
| Chronic hypertension - | 1.00 (0.97-1.04) |  |
| Vasopressor + | 1.01 (0.99-1.04) | 0.78 |
| Vasopressor - | 1.00 (0.97-1.03) |  |
| Time spent with MPP-deficit >20%, hour |  |  |
| Overall | 1.01 (0.99-1.04) |  |
| CPB + | 1.02 (0.995-1.05) | 0.030 |
| CPB - | 0.94 (0.86-1.04) |  |
| Chronic hypertension + | 1.01 (0.98-1.04) | 0.95 |
| Chronic hypertension - | 1.01 (0.96-1.03) |  |
| Vasopressor + | 1.01 (0.98-1.05) | 1.0 |
| Vasopressor - | 1.01 (0.97-1.04) |  |
| Time spent with MAP <65 mmHg, hour | | |
| Overall | 1.02 (0.99-1.06) |  |
| CPB + | 1.03 (0.99-1.07) | 0.23 |
| CPB - | 0.99 (0.84-1.17) |  |
| Chronic hypertension + | 1.01 (0.97-1.06) | 0.50 |
| Chronic hypertension - | 1.05 (0.97-1.13) |  |
| Vasopressor + | 1.02 (0.96-1.08) | 0.64 |
| Vasopressor - | 1.02 (0.97-1.07) |  |
| Time spent with MAP-deficit >20%, hour |  |  |
| Overall | 1.01 (0.98-1.03) |  |
| CPB + | 1.02 (0.99-1.05) | 0.027 |
| CPB - | 0.92 (0.82-1.03) |  |
| Chronic hypertension + | 1.01 (0.98-1.04) | 1.00 |
| Chronic hypertension - | 1.01 (0.95-1.06) |  |
| Vasopressor + | 1.00 (0.96-1.04) | 0.46 |
| Vasopressor - | 1.01 (0.98-1.05) |  |

Adjusted on age, APACHE II score, chronic hypertension, preoperative left ventricular ejection fraction, baseline serum creatinine, surgery time, cardiopulmonary bypass, intraoperative fluid balance, and postoperative transfusion within 24 hours after ICU admission.

OR: odds ratio; CI: confidence interval; AKI: acute kidney injury; ICU: intensive care unit; MAP: mean arterial pressure; CVP: central venous pressure; MPP: mean perfusion pressure; CPB: cardiopulmonary bypass

# Figure S1: Details of data collection

**
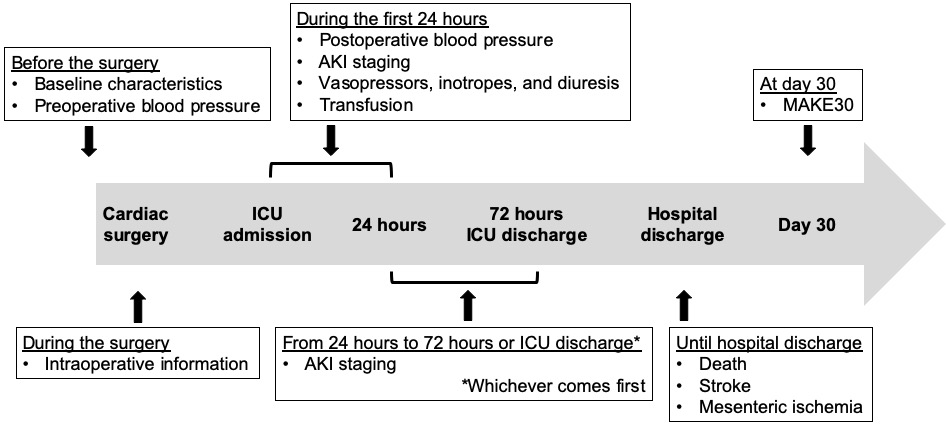
**

ICU: intensive care unit; AKI acute kidney injury; MAKE: major adverse kidney event.

#

# Figure S2: Patient flow diagram


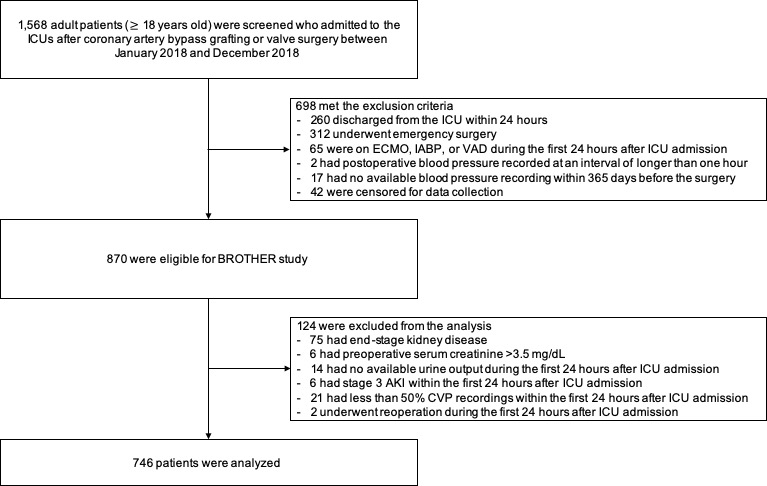


ICU: intensive care unit; IABP: intra-aortic balloon pumping; ECMO: extracorporeal membrane oxygenation; VAD: ventricular assist device; AKI: acute kidney injury; CVP: central venous pressure

# Figure S3: Achieved MAP (a) and MAP-deficit (b) during the first 24 hours after ICU admission

(a)

(b)

MAP: mean arterial pressure; ICU; intensive care unit; AKI: acute kidney injury
